# An Equity-Focused Assessment of Evidence-Based Parenting Intervention Research

**DOI:** 10.1007/s10567-024-00479-2

**Published:** 2024-05-16

**Authors:** Suzanne E. U. Kerns, Samuel J. Maddox, Ruth E. Berhanu, Heather Allan, Rachel A. Wilson, Antonia Chiesa, Rebecca Orsi-Hunt, Lauren Pryce McCarthy, Lesly J. Henry, Chaundrissa Oyeshiku Smith

**Affiliations:** 1 The Kempe Center for the Prevention and Treatment of Child Abuse and Neglect, Department of Pediatrics, School of Medicine, University of Colorado Anschutz, Aurora, USA; 2 Department of Psychology, Clayton State University, Morrow, USA; 3 The Southeast Permanente Medical Group, Atlanta, USA

**Keywords:** Parenting, Racial equity, Culturally responsive evaluation, Research methods

## Abstract

Evidence-based parenting interventions (EBPI) support children and families to promote resilience, address emotional and behavioral concerns, and prevent or address issues related to child maltreatment. Critiques of EBPIs include concerns about their relevance and effectiveness for diverse populations when they are implemented at population scale. Research methods that center racial equity and include community-based participatory approaches have the potential to address some of these concerns. The purpose of the present review was to document the extent to which methods associated with promoting racial equity in research have been used in studies that contribute to the evidence base for programs that meet evidentiary standards for a clearinghouse that was developed to support the Family First Prevention Services Act in the United States. We developed a coding system largely based on the Culturally Responsive Evaluation model. A sample of 47 papers that are part of the evidence base for ten in-home parent skill-based programs were reviewed and coded. Only three of 28 possible codes were observed to occur in over half of the studies (including race/ethnicity demographic characteristics, conducting measure reliability for the study sample, and including information on socioeconomic status). Although the overall presence of equity-informed methods was low, a positive trend was observed over time. This review highlights ways in which rigorous research can incorporate racial equity into the planning, design, execution, and interpretation and dissemination of programs of study. We posit that doing so improves the external validity of studies while maintaining high-quality research that can contribute to an evidence base.

**Supplementary Information:**

The online version contains supplementary material available at 10.1007/s10567-024-00479-2.

Concerns have been raised about how to increase the relevance of evidence-based parenting interventions (EBPIs) for diverse populations, particularly in the United States (Doyle et al., 2023; Weisenmuller & Hilton, 2021). This paper focuses on the question “to what extent do EBPIs incorporate features of racial equity in their study designs?” While one important approach is to increase the relevance of the intervention design (e.g., culturally specific adaptations of existing programs or creating new programs for specific populations), another important consideration, and the focus of this paper, is how to ensure that the research that contributes to the evidence base is rooted in principles of racial equity (Andrews et al., 2019; Shapiro et al., 2024). This paper is written in honor of the contributions that Drs. Ronald J. Prinz and Thomas Ollendick have made over the past several decades to increase the research base for treatments for children and parenting supports, including how to ensure the relevance of such programs across diverse populations. Three of the authors of this paper (Drs. Suzanne Kerns, Samuel Maddox, and Chaundrissa Oyeshiku Smith) were doctoral students of Dr. Prinz in the late 1990s-2000s.

## Background

Although a plethora of evidence-based and empirically supported interventions to improve public health outcomes exist, the US still lags most industrialized countries in reducing health disparities (Biglan et al., 2023). According to the US National Center for Health Statistics (Dean & Fenton, 2023), health disparities are inequities in health outcomes influenced by economic, sociodemographic, and environmental and historical determinants. These determinants such as poverty/lack of resources, limited access to high-quality health care, adverse life experiences, systems of discrimination, and oppression among many others have led to lower life expectancy and increase in mortality rates, disability, and mental illness for disadvantaged populations (Ahmad et al., 2023; APA, 2019; Biglan et al., 2023; Dean & Fenton, 2013). Though the reasons for these determinants are complex and have deep historical roots, one critique of EBPIs is that they do not adequately consider or address these determinants and thus have questionable social valence despite having research evidence of effectiveness (Chicago Beyond, 2019).

A recent United States-based surveillance study found significant prevalence differences by race for child mental and emotional behaviors, for example, Black and White children had higher rates of ADHD compared with Hispanic and Asian children, and Black children were more likely than other racial/ethnic groups to be diagnosed with behavior/conduct problems (Bitsko et al., 2022). Considering treatment, less than half of children and youth with mental health needs receive any form of treatment (Whitney & Peterson, 2019), and considering evidence-based treatments (EBTs) specifically, it remains extremely uncommon for children and youth to access EBTs at a population level. One study found very low penetration rates (only 1–3% of the eligible population) for EBTs for children and youth (Bruns et al., 2016). One hypothesis for the stagnant progress in addressing health disparities is that, although there has been an increased focus on culture and cultural context, the practical application of these concepts to the planning, development, implementation, and evaluation of interventions to address health disparities may be lacking (Andrews et al., 2019; Hood et al., 2015). Based on this hypothesis, it is then incumbent upon program developers, funders, researchers, evaluators, practitioners, and their associated agencies to utilize more equitable and culturally adaptive frameworks if we are to have a lasting positive impact on health outcomes for disadvantaged populations.

Parenting programs are particularly relevant for children and youth involved in or at risk for involvement in the child welfare system (Prinz, 2019). Dr. Prinz and colleagues call for population-based approaches to supporting parents and reducing risks for maltreatment (Prinz, 2019; Sanders, 2019). To do this well, there must be consideration of the diverse needs of different families and communities. In addition to reviewing studies of parenting interventions for inclusion of racial equity-informed research methods, the current paper provides a coding scheme that can be used to determine the extent to which principles relevant to racial equity went into study planning, development, implementation, and evaluation. We pilot tested this coding scheme with parenting EBPIs that are likely to be considered for implementation in response to a US-based federal initiative called the Family First Prevention Services Act (FFPSA, 2018). The FFPSA legislation aims to reduce the use of foster care by incentivizing the provision of prevention and treatment programs and services to families before out-of-home placements are recommended. It represents a substantial reconstruction of the current child welfare system (Villalpando, 2019). The legislation specifically incentivizes use of mental health, in-home parent skill-based, substance abuse, and kinship navigator programs and services.

### Contributions of Ron Prinz to Research on EBPIs and Considerations of Race Equity

The specific focus on parenting interventions was chosen due to the prolific work of Dr. Ronald Prinz and his mentorship of several authors on this paper. For several decades, Dr. Prinz has engaged in extensive prevention science research focusing on reducing risk factors and promoting protective factors in youth. In addition, through numerous grants, he has trained and mentored doctoral students in psychology, including authors on this paper, to become prevention scientists and clinicians working with youth from diverse populations. As the founder of the University of South Carolina Research Center for Child Well-Being, Dr. Prinz continues his service to youth and their families. Throughout his work and mentorship, Ron has maintained a focus on racial equity as a necessary component to any youth intervention or parenting program (Kerns & Prinz, 2002; Prinz, 2019). It is with this guidance that the authors of this paper present an equity-focused assessment of evidence-based parenting intervention research.

### Significant Initiatives Influencing Racial Equity in EBPIs

#### Offices of Minority Health Initiative

To address the issue of health disparities, former US Health and Human Services Secretary, Margaret Heckler, established the Offices of Minority Health. As part of the initial comprehensive report (Heckler, 1985), seven recommendations emerged. These recommendations included (1) develop an outreach campaign to promote access to information in diverse communities using culturally appropriate education materials, (2) ensure culturally responsive patient education within healthcare settings, (3) innovate the delivery and financing of culturally acceptable healthcare services, (4) collaborate with government agencies, health departments and organizations, institutes of higher education, and the public and private sector to develop healthcare strategies for diverse populations, (5) engage in intra-governmental coordinated efforts to ensure effective healthcare strategies for diverse populations, (6) enhance the capacity of state, local and community constituents to meet the health needs of diverse populations, and (7) increase utilization of existing data and improvements in future data collection to enhance opportunities for the analyses of the unique health needs of diverse populations (Heckler, 1985).

As part of these recommendations, healthcare providers, agencies, and researchers had a blueprint for how to develop programs that could address the racial equity gap and health disparities in general. However, a close review of the recommendations still reveals a limited perspective in implementing and evaluating the cultural equity of a program. Specifically, the evidence presented in the report supporting the recommendations often only focused on surface-level cultural equity issues such as language translation or oversampling. These are strategies that function to include more diverse persons but fall short of substantive considerations of needs for cultural or contextual adaptations (Heckler, 1985). In addition, the evidence sometimes used deficit-focused and stigmatizing language such as suggesting behavior modification and anger management to reduce homicide in the African American community (Heckler, 1985). While these and similar efforts can address some of the putative factors of community violence, a more comprehensive prevention approach is warranted. Therefore, this initiative, although groundbreaking, fell short in addressing the problems of health disparities.

#### American Psychological Association Guidelines for Multicultural Practice

The American Psychological Association’s (APA) Guidelines for Multicultural Practice (APA, 2002) represents the landmark effort for equity in the practice of behavioral healthcare. APA is the leading professional body and accrediting organization for the training and practice of psychology in the United States (APA, 2022). It provides guidance in areas such as education, mental health, ethics, diversity, equity and inclusion for practitioners, students, training programs, researchers, and interventionists. One of the major areas of guidance from APA that has evolved over the last few decades is multiculturalism and diversity. Multiculturalism can best be understood as the influence of demographic factors such as age, race, ethnicity, gender, sexual orientation, ability status, religious orientation, socioeconomic status, primary language, country of origin, and others on the development of a person’s identity and their interaction with the world (APA, 2002).

Early attempts from APA at providing multicultural guidance existed in the 1990s (APA, 1990). APA later officially proposed guidelines for multicultural practice in 2002 and updated them in 2017. The original multicultural practice guidelines emphasized that psychologists be aware of their own biases, incorporate multiculturalism in training and education, and support culturally informed policy development and practices. Specific to research, these guidelines specified that psychologists should use culturally informed and ethical research practices for diverse populations. This guideline applies to the generation of research ideas, through the design phase, the assessment phase, and analyses and interpretation (APA, 2002). In 2017, greater specificity was added to recommendations, including highlighting the importance of the role of community contexts, acknowledging the fluidity of identity, and enhancing the understanding of issues associated with power, privilege, and oppression. The 2017 guidelines retained the emphasis that research be culturally appropriate and informed (APA, 2017).

Importantly, in addition to the guidelines, APA has also acknowledged its contribution to historical injustices regarding multiculturalism and diversity in research and practice (APA, 2021). Such injustices include psychological research that led to racially disproportionate incarceration, harmful treatments for LGBTQI + identifying individuals, and gender segregation.

#### Executive Order 13,985

On January 20, 2021, US President Joseph Biden signed Executive Order 13,985. This executive order titled *Advancing Racial Equity and Support for Underserved Communities Through the Federal Government* lays out a federal policy aimed at advancing racial equity and promoting support for underserved communities within the United States. This executive order acknowledges that equal opportunity is fundamental to American democracy and emphasizes the strength of the country's diversity. The policy calls for a whole-of-government equity agenda that matches the scale of the challenges faced by the nation. The key elements of this order include conducting equity assessments; coordinating and collaborating within government and with external groups including communities; collecting and analyzing data along racial, ethnic, and other demographic factors to assess racial equity; prioritizing equity considerations in policy; supporting underserved communities; enhancing diversity, equity, and inclusion within the Federal workforce; distributing resources in ways that address disparities; and encouraging community engagement. The executive order represents a policy shift aimed at addressing systemic inequities and promoting inclusivity within the United States through the federal government's actions and policies (Exec. Order No. 13985, 2021).

### Empirically Supported Parenting Programs and Racial Equity Considerations

These initiatives, whether to promote equity in federal services (Exec. Order No. 13985, 2021), healthcare in general (Heckler, 1985), or behavioral healthcare (APA, 2002), all provided direction to the field around research, data collection and evaluation. It is of interest to see how the field has adapted to and evolved over time because of these guidelines. Because of the focus of this special issue, we chose to examine the evolution of racial equity in research by examining the empirical literature specific to parenting programs. Parenting programs are especially relevant to the population of children and their families who are at risk for or are involved with the child welfare system. Coercive or punitive parenting practices are associated with child maltreatment (Azar, 2002; Rodriguez, 2010), and giving parents or caregivers skills to meet the emotional and behavioral needs of children and youth supports the well-being objective of the US child welfare system (Horwitz et al., 2010).

Parenting programs are typically identified as programs that include some form of parent/caregiver education and/or training. The value of parenting programs to address youth emotional and behavioral concerns has been well established over the past decades (Prinz & Sanders, 2007). Many of these programs have gained significant empirical support (e.g., Parent Child Interaction Therapy [Funderburk & Eyberg, 2011], Helping the Noncompliant Child [McMahon & Forehand, 2003], Triple P Positive Parenting Program [Sanders, 1999], The Incredible Years [Webster-Stratton, 2005]). However, there are concerns regarding the application of these empirically supported and evidence-based programs to diverse and/or underserved populations (Baumann et al., 2015; Domenech Rodriguez et al., 2011; Lau, 2006). Although some research supports a universalistic perspective where the program can be implemented with fidelity across diverse populations and still have positive outcomes (Chaffin et al., 2012; Huey & Polo, 2008), there has been a significant push for cultural adaptation of empirically supported treatments. However, there are arguments that many such cultural adaptations consist only of minor and superficial modifications such as language translation or altering examples or pictures. While some programs have been subject to deeper cultural adaptation techniques considering values and belief systems of the populations targeted for adaptation (Bernal et al., 1995; Domenech Rodriguez et al., 2011; Hwang, 2006), the research and evaluation into these programs appear to suffer from similar difficulties translating race equitable research from theory to practice (Boyce, 2017). These criticisms mirror the limitations of Heckler’s (1985) minority health initiative.

### Race Equity in Research Best Practices

According to *Executive Order 13,985*, equity can be defined as systematic treatment of all individuals including those in underserved populations in a just, fair, and impartial manner. There are multiple ways equity can occur within a research study or program evaluation that elevate the role of the individuals and communities who are being served and are the focus of the research. In the planning and design phase, equity-driving research engages the target population in defining the problem and need, identifying targets for intervention, and selecting the interventions themselves (Andrews et al., 2019; Kien, 2007). In the study implementation phase, equity can be impacted based on engagement and recruitment of participants, retention efforts, and approaches to program delivery (Chicago Beyond, 2019; Langer et al., 2021). The equity relevance of a program’s research base can be influenced by the evaluation process including what data are collected, from whom, by whom and how. In addition, the ability to understand equity in program evaluation is affected by how data are analyzed, interpreted, represented, and disseminated; indeed, an equitable evaluation solicits the feedback from beginning to end, engaging participants in the selection of research questions through the interpretation of findings to ensure that the results are meaningful to those whose programs purport to serve (Chicago Beyond, 2019; Sevak et al., 2022).

For our assessment of racial equity approaches within parenting program research, we leaned heavily on Hood and colleagues' Culturally Responsive Evaluation Model (CRE; Hood et al., 2015). This model attempts to address limitations in traditional evaluation frameworks such as superficial inclusion of cultural concepts and lack of clarity regarding consequential engagement (Boyce, 2017). Hopson (2009) describes CRE as a theoretical and political framework attuned to culture during the various phases of research from design to dissemination offering a meaningful and systematic lens to examine the incorporation of equity-related principles in the evaluation of parenting programs.

CRE contains nine procedural stages that the authors deem necessary to establish decolonizing epistemologies that critically evaluate the role of culture and promote social advocacy in the evaluation process (Hood et al., 2015; Hopson, 2009). These include preparation, engagement, purpose, framing questions, design, instrumentation, data collection, data analysis, and dissemination. In each of these procedural stages, recommendations are made to ensure racial equity. Specific recommendations are detailed below.

During research preparation, the researchers or evaluators should become familiar with the community, including their stories, history, geographical influence, power distribution, relevant factors associated with diversity, and how people within the community relate to others, including those conducting research. During research engagement stages, consideration is given to how trust is built and what strategies are used to engage with community representatives. Other examples include how researchers collaborate with community partners in establishing the purpose of the research, including any potential impacts associated with changes to balance of power. This framework advocates for community partners to be involved in identifying the research questions and defining what constitutes credible evidence. Evaluation design should include consideration to what type and how data are collected. Measures should have multicultural validity defined as "the accuracy or trustworthiness of understandings and judgments, actions, and consequences, across multiple, intersecting dimensions of cultural diversity" (Kirkhart, 2010, p. 401), in recognition that all human experiences occur in context and are inherently subjective. Thus, attending to how items are interpreted across various populations toward establishing cultural validity is critical. Further, attention should be paid to how data are collected, including how, from, and by whom; Hood and colleagues (2015) posit that community partners and, if relevant, cultural interpreters (e.g., members of the community of study who help translate concepts), actively participate in data analysis and interpretation. Finally, dissemination products should be accessible, culturally responsive and focus on the benefits to the community, including social justice considerations (Hood et al., 2015). Despite this guidance, a scoping review found that even among 52 articles that specifically examined cultural responsivity and touted applying the CRE framework, there were inconsistencies in how CRE was incorporated across studies (Kushnier et al., 2023).

For the purposes of this review, we leaned heavily on CRE-based recommendations to formulate our codes used to describe racially equitable research methods. Distinct from the Kushnier et al. (2023) scoping review, we applied this framework to studies of evidence-based parenting interventions that are eligible for dissemination through FFPSA. Of note, the studies in our review were not explicitly designed using a CRE framework. Rather, our study sought to examine the degree to which existing evaluations of parenting programs included best practices for being culturally responsive. This is important because such programs are likely to be implemented in a diverse array of jurisdictions with a high proportion of vulnerable and marginalized populations receiving child welfare services.

### Study Context: Parenting Programs on the Title IV-E Prevention Services Clearinghouse

For this first attempt at coding the racial equity-related features of research that informs the evidentiary rating of parenting interventions, we wanted to ground the work in a meaningful policy context. We narrowed the inclusionary criteria of parenting programs rated by the Title IV-E Prevention Services Clearinghouse to both align with the focus of the special issue and because of the policy relevance of programs included on the registry. This Clearinghouse was developed in response to the US-based Family First Prevention Services Act (FFPSA) of 2018. While this legislation has many facets, one key feature is an effort to direct funding for programs and services rated as well-supported, supported, and promising toward preventing the need for foster care placements and/or addressing targeted risk factors that increase likelihood of child welfare involvement for families. The Clearinghouse reviews the evidence base for these programs and services and provides ratings to inform state plans for FFPSA implementation (Wilson et al., 2019). For the purposes of this study, we examined in-home parent skill-based programs that were targeted to address child behavioral health outcomes and previously reviewed and rated by the Clearinghouse. Our goal was to explore the extent to which these studies used best practices in culturally responsive and race equitable research methodology. While we hope to illuminate the current state of research on EBPs for parenting interventions, highlight gaps in prior research, and promote improvements in race equity analysis for future studies, we also hope to present a coding framework that can be utilized to assess bodies of evidence for beyond parenting programs, given that the principles of CRE are not limited in their application to any specific program type.

## Method

The coding methods were informed by multiple sources identified through a literature search. Several manuscripts and gray literature sources were consulted to determine the priority codes for this paper, including Chicago Beyond (2019), Hawn et al. (2022), and Hood et al. (2015).

For the purposes of this review, a *program* is defined as a prevention or intervention program or service that has a distinct manual. A *study* is defined as “one research investigation of a defined subject sample, and the interventions, measures, and statistical analyses applied to that sample” (Wilson et al., 2019, p. 9). A *paper* is defined as a manuscript or report that contains information about the identified study. One or more papers may be part of a study, as it is common for study authors to publish multiple papers reporting various findings associated with a single study.

### Article Identification

**Fig. 1:**
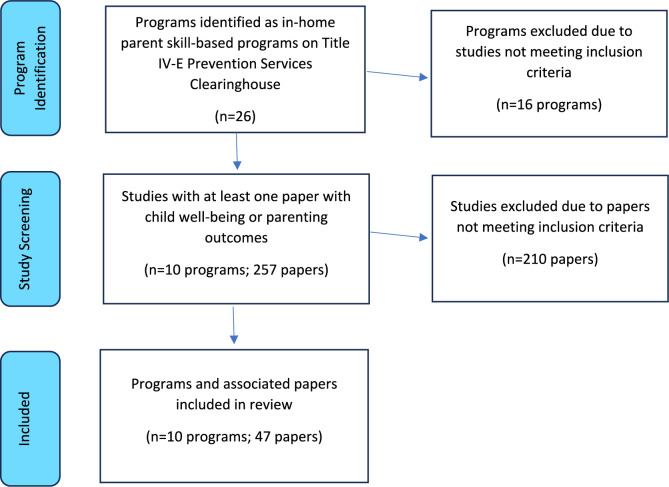
Adapted PRISMA flow diagram (Moher et al., 2009)

The Title IV-E Prevention Services Clearinghouse website (https://preventionservices.acf.hhs.gov/) was consulted to identify the papers that met inclusionary criteria. The inclusionary criteria occurred at two levels—the program and study level. For the program level, articles must (1) target a program that was rated in the Clearinghouse as of July 11, 2023, within the “in-home parent skill-based” program area and (2) were rated as either well-supported, supported, or promising. At the study level, (3) individual studies had one or more papers with a target outcome of either (a) child well-being: behavioral and emotional functioning or (b) adult well-being: positive parenting practices; and (4) studies must contribute to the evidence base for the program, meaning the study must have a rating as high or moderate in design and execution standards, as defined by the Clearinghouse (Wilson et al., 2019). In total, 47 papers (representing 23 studies) from ten programs and services met inclusionary criteria (see Fig. 1).

### Data Extraction and Coding

Papers were downloaded in full and shared across the entire coding team. All papers were independently double coded, and codes were recorded in a Qualtrics survey. After individual coding completion, coding pairs met to address any discrepancies and come to consensus. One coder had to drop out of the study part way through coding completion. For this coder, the first author arbitrated coding. An initial training meeting and two subsequent group meetings were held to discuss challenging codes and ensure consistency in coding. Initial rating agreement at the paper level (prior to arbitration) ranged from 34.0 to 97.8%. The one code with only 34% agreement was indicating if White race (the default dominant culture in the United States) was used as a comparison group. Many of the papers in our sample were from countries outside of the United States with different demographic and cultural contexts. As such, the coding team determined that code was not practical to consistently interpret in an international context and would be dropped from the final analyses. After that code was dropped, the lowest percent agreement by code was 66%. After consensus meetings, 100% interrater reliability was achieved for all codes.

### Data Coding Manual and Synthesis

The coding manual is available in Supplemental Appendix 1. All codes were either 0 = no, 1 = yes or N/A = not applicable. N/A codes were used when papers did not report on participant race/ethnicity at all precluding coders from assessing *how* race/ethnicity was cared in various domains throughout the study. Coders were instructed to err on the side of inclusion. That is, if there was any evidence that a coding domain was present in the paper, even if it was not a ‘perfect’ example of the domain, to code as a 1, and then leave detailed notes concerning domains that were difficult to code. Initial coding occurred at the paper level, so that all papers that met inclusion criteria were independently double coded separately and then, after reconciliation was combined at the study level whereby if any paper within a study had included a coding domain, the study received a “1” for that domain. Finally, codes were rolled up to the program level, such that if any study for the program included a particular racial equity domain, the program received a “1” for that domain.

#### Program Level Codes

Background information for each program was collected (Table 1), including the primary target age range for the program, whether it was a prevention or treatment program, the priority target populations (e.g., parents, mothers, teens), available languages, the Clearinghouse evidentiary support rating (well-supported, supported or promising), the total number of studies that constituted the evidence rating, and the total number of studies that were reviewed by the Clearinghouse, including whether the number of studies exceeded ten, because, for programs with more than ten eligible studies, not all studies are evaluated by the Clearinghouse which could therefore impact the accuracy of our study.

**Table 1:** Title IV-E prevention services clearinghouse program/service demographics

Program Name	Study #^a^	Author (Year)	Age Range	Prev. or Treat	Program Focus	Parent/ Caregiver Status	Language	Clearinghouse Rating	Good Study Count	Total Study Count	Study Count > 10
Brief Strategic Family Therapy	10,570	Horigian et al. (2013)	6–16	T	Substance abuse, mental health disorders, family dysfunction, delinquency	Families with maladaptive interactions	E, Sp	W-S	1	5	No
		Horigian et al. (2015a)									
		Horigian et al. (2015b)									
Child First	11,295	Lowell et al. (2011)	0–5	P	Prevent or diminish serious child social-emotional disturbance, behavioral problems developmental and learning abilities, and abuse and neglect	N/A or Not Specified	E, Sp	S	1	2	No
Familias Unidas	11,791	Pantin et al. (2009)	12–16	T	Prevent substance abuse, risky sexual behavior among Hispanic adolescents, parenting skills, parent–adolescent communication, parental involvement, and investment in adolescents	Families of Hispanic adolescents aged 12–16	E, Sp	W-S	4	4	No
		Perrino et al. (2016)									
	11,796	Lee et al. (2019)									
	11,797	Prado et al. (2012)									
	11,798	Molleda et al. (2017)									
Family Check-Up	11,074	Gardner et al. (2009)	2–17	T	Child: Behavior problems, social and emotional adjustment, emotional distress, self-regulation and school readiness/school attendance and grades, depression (adolescents), antisocial behavior/delinquent activity. Parent: Positive parenting, coercive conflict, monitoring (adolescence). Dyad: Parent–adolescent conflict; parenting skills and family management practices, with the goals of improving a range of emotional, behavioral, and academic child outcomes	Parents	E, Sp, O	W-S	4	5	No
		Lunkenheimer et al. (2008)									
		Shaw et al. (2009)									
		Shelleby et al. (2012)									
		Shelleby et al. (2018)									
		Smith et al. (2014)									
		Smith et al. (2019)									
		Wang et al. (2019)									
	11,082	Hiscock et al. (2018)									
	11,083	Shaw et al. (2006)									
	11,093	Stormshak et al. (2020a)									
		Stormshak et al. (2020b)									
Generation PMTO- Individual	11,328	Scavenius et al. (2020)	2–17	T	Parents of children with behavioral problems such as aggression, antisocial behaviors, conduct problems, oppositional defiance, delinquency, and substance use; child behavior problems such as oppositional behavior, conduct problems, substance use, delinquency, aggression, and antisocial behaviors	Parents and caregivers of children with behavioral problems	E,Sp,O	Pr	2	7	No
	11,330	Sigmarsdóttir et al. (2013)									
		Sigmarsdóttir et al. (2015)									
Healthy Families America	10,137	Duggan et al. (1999)	0–2	P	Increased risk for maltreatment or other adverse childhood experiences; Intervention sites may choose to target low-income families, single parent households, or families who have experienced substance use, mental health issues, or domestic violence	Each HFA site is able to determine which family and parent characteristics it targets. PC specifies parents	E, Sp	W-S	4	22	Yes
		El-Kamary et al. (2004)									
		Duggan et al. (2004a)									
		Duggan et al. (2004b)									
	10,138	DuMont et al. (2008a)									
		DuMont et al. (2008b)									
		DuMont et al. (2010)									
	10,141	Caldera et al. (2007)									
		Duggan et al. (2005)									
	10,250	LeCroy et al. (2011)									
Multidimensional Family Therapy	10,644	Henderson et al. (2009)	9–18	T	Substance use, delinquency, mental health, academic/vocational, and emotional problems	N/A or Not Specified	E, Sp, O	S	2	2	No
		Liddle et al. (2004)									
		Liddle et al. (2009)									
	10,649	Schaub et al. (2014)									
Nurse Family Partnership	10,167	Olds et al. (2002)	0–2	P	To improve the health, relationships, and economic well-being of mothers and their children; healthy pre and postnatal development	N/A or Not Specified	E, Sp	W-S	1	10	No
		Olds et al. (2004)									
		Olds et al. (2014)									
Promoting First Relationships	14,671	Oxford et al. (2021)	0–5	P	Child social-emotional development, child-caregiver trust, child and caregiver emotional regulation and self-reflection, caregiver challenging behaviors	Parents, grandparents, caregivers with a mental health diagnosis, childcare teachers, adolescent mothers, first-time parents, foster parents	E, Sp, O	S	1	9	No
Video Interaction Project	14,798	Mendelsohn et al. (2007)	0–5	P	Child social-emotional, cognitive and language growth	N/A or Not Specified	E	Pr	3	3	No
	14,799	Cates et al. (2018)									
		Mendelsohn et al. (2018)									
	14,803	Weisleder et al. (2016) Roby et al. (2021)									

^a^Study number corresponds to the assigned study number in the Title IV-E Prevention Services Clearinghouse

P = prevention, T = treatment; E = English, Sp = Spanish, O = Other; W-S = Well-supported, S = Supported, Pr = Promising. We used the language used in the study to describe program needs and parent/caregiver status

#### Paper Level Codes

*Paper descriptive information.* Individual papers were coded as to the type of document (peer-reviewed manuscript, US-based governmental report, non-US-based governmental report, commissioned report, and other), the type of study (randomized control trial or quasi-experimental design), the Clearinghouse-eligible target outcome domains that were included, and the data source (child, parent/caregiver, or teacher self-reports; administrative, observational, physiological, and other).

*Racial and Ethnic Equity Domains.* Individual papers were coded by racial and ethnic equity domains. Domains were organized by four stages of research: study planning and development, methods and analysis planning, reporting, and interpretation (please see Supplemental Appendix 1 for a full list of codes within each domain).

*Other.* Coders additionally noted any domains that were difficult to code, if study the authors specified a racial equity-informed framework, and any other notes pertaining to coding.

### Coding Team

Eight authors were coders and all co-authors engaged in data interpretation. The entirety of the team were multidisciplinary (psychology, social work, pediatrics, public health, epidemiology), multiracial, and diverse in age and gender. Together, we strived to have mutual accountability and consider multiple perspectives while designing our approach and the coding manual, and while analyzing and interpreting the results. Despite this, we acknowledge that our own experiences and positionality may have influenced aspects of this study, as they do in any study. We welcome critical dialogue and constructive feedback on our approach and the potential for introduction of bias in the many forms it may take.

## Results

### Descriptive Results

Table 1 provides descriptive information about each program and service included in the review. On average, there were 4.8 papers per program (range: 1–12). The programs represented a mix of prevention (n = 5) and treatment (n = 5). One program, Familias Unidas, was developed specifically for the Latinx population, and Healthy Families America had multiple sites that served different racial/ethnic populations; the rest did not specify a racial or ethnic target population. Nine of the ten programs were available in languages other than English, with Spanish being available for all nine and additional languages available in four. Five of the reviewed programs met the “well-supported” FFPSA evidentiary threshold (Brief Strategic Family Therapy, Familias Unidas, Family Check-Up, Healthy Families America, and Nurse Family Partnerships), while three met “supported” (Child First, Multidimensional Family Therapy, Promoting First Relationships) and two met “promising” (Generation PMTO-Individual and Video Interaction Project). Only one program (Healthy Families America) had more than ten studies that could have been reviewed by the Clearinghouse. When this happens, the Clearinghouse starts with the first ten studies and continues to review studies to ensure the highest possible rating. Studies not included in the rating are still assessed for risk of harm but are not included in the evidence base for the program rating. Thus, for this program, we only reviewed the ten studies that were included for the Clearinghouse rating determination.

### Racial Equity Strategies Observed in Studies and Programs

Table 2 displays the frequencies at which the racial equity codes were observed across papers and programs. In the planning/study development domain, the most frequently observed code was “research questions/s were directly related to racial equity,” which was observed in 27.1% of the papers and 60% of the programs coded. In this domain, the least frequently observed code was any inclusion of community-based or youth-based participatory research (CBPR/YBPR) or community inclusion methods, which only occurred within two (4.1%) of papers and two programs (20%), Healthy Families America and Familias Unidas.

**Table 2:** Extent of racial equity strategies used by paper and by program

	# (%) papers observed^a^	# (%) programs observed (N = 10)
Research Planning/Study Development		
Included CBPR/YBPR or other community engagement techniques	2 (4.1%)	2 (20%)
Research question/s were directly related to racial equity	13 (27.1%)	6 (60%)
Evaluators were trained in best practices for working with the population of focus	3 (6.3%)	3 (30%)
Described why or how race/ethnicity is defined for the purposes of the study	10 (22.7%)	4 (40%)
Methods, Analysis Planning		
Used mixed methods or stories as part of the analyses	0 (0%)	0 (0%)
Specifically conducted race equity analyses	9 (20.5%)	3 (30%)
Participants recruited in ways that were likely to include representative members of the population of focus	15 (31.3%)	6 (60%)
Engaged in retention efforts with the intention of reducing disproportionate attrition	1 (2.2%)	1 (10%)
Used cognitive interviewing or pilot testing of measures to understand how different people interpret questions	0 (0%)	0 (0%)
Conducted validation or reliability metrics for the study sample	29 (72.5%)	9 (90%)
Purposefully oversampled populations to avoid making conclusions based on small sample sizes	8 (16.7%)	4 (40%)
Study included features of place (e.g., census block, zip code) in examining outcomes	7 (14.6%)	4 (40%)
Variables included items related to perceptions of discrimination or oppression	0 (0%)	0 (0%)
Data Reporting		
Included race/ethnicity in demographic characteristics	44 (91.7%)	10 (100%)
Participants were able to self-describe their race-ethnicity (i.e., there were not pre-populated choice options)	1 (2.3%)	1 (10%)
Participants were able to self-describe their gender (i.e., there were not pre-populated choice options)	0 (0%)	0 (0%)
Grouped racial/ethnic groups with small n together as ‘other’*	20 (50%)	7 (70%)
CONSORT or other description of how participants moved through the study included race/ethnicity information	3 (6.8%)	2 (20%)
Provided results separately by race or ethnicity	3 (6.8%)	2 (20%)
Assessed for differential attrition by race/ethnicity	16 (38.1%)	5 (50%)
Included subgroup analyses by race/ethnicity and/or other areas of intersectionality to assess what works for whom	5 (11.4%)	3 (30%)
Included information on socioeconomic status of program participants	44 (91.7%)	10 (100%)
Reported on the acceptability of the intervention to participants, disaggregated by race/ethnicity	2 (4.4%)	1 (10%)
Analysis plan included examining intersectionality of race with other dimensions of identity on study outcomes	1 (2.2%)	1 (10%)
Conclusions and Interpretation of Results		
Included community groups to support data interpretation	0 (0%)	0 (0%)
Indicated limitations associated with racial equity-related considerations	17 (38.6%)	5 (50%)
Acknowledged the potential impact of structural bias	2 (4.5%)	2 (20%)
At least one of the conclusions drawn by the paper focused on a feature of diversity, inclusion, or equity	16 (33.3%)	7 (70%)

Most codes can be interpreted positively (the presence of this feature indicates consideration to racial equity). Codes with an asterisk (*) are to be interpreted negatively (presence of this feature is not consistent with best practices for racial equity)

^a^If a paper did not include any information about race/ethnicity, many codes were N/A. Thus, there are slightly different denominators calculating the percentages

Overall, racial equity codes associated with methods and analysis planning were not well represented. The most frequently used strategy was to evaluate psychometric properties of measures using the study sample. This was observed across 72.5% of papers and all but one (90%) program. Three codes within this domain were not observed at all: mixed methods or stories, cognitive interviewing, and inclusion of variables related to perceptions of discrimination or oppression.

More frequently observed were racial equity-associated approaches within the reporting domain. Within this domain, all programs reported information on race/ethnicity and socioeconomic status of the study sample. However, only one study (of Promoting First Relationships) allowed participants to self-describe their race/ethnicity, in accordance with CRE best practices, and none allowed for self-described gender. Papers rarely disaggregated results by race/ethnicity (Brief Strategic Family Therapy and Family Check-Up were programs that did have papers that disaggregated) or considered intersectionality of race with other social identities when examining program effectiveness (one paper under Video Interaction Project). Against CRE best practice recommendations, many of the reviewed papers consolidated smaller racial populations into an “other” category for the purposes of data reporting; this practice was done in about 50% of the papers reviewed.

Finally, we examined the extent to which racial equity was considered in paper conclusions and interpretation of findings. On the low end, none of the papers reviewed included community groups or study participants in the interpretation of study findings. On the higher end, 38.6% of papers and 50% of programs discussed study limitations associated with racial equity-related considerations; 33.3% of papers across 70% programs discussed racial equity-related conclusions.

### Observed Changes Over Time

**Fig. 2:**
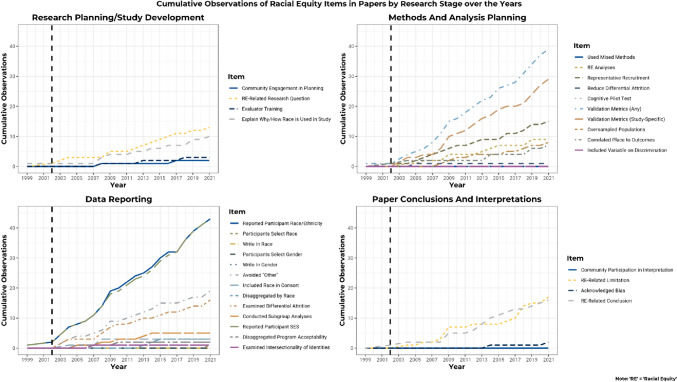
Cumulative observations of racial equity (RE) over time

We wondered whether there have been improvements over time in use of racial equity-related research methods. Figure 2 shows a slight upward trend for cumulative observations of each of our racial equity codes over time. This figure shows only minimal changes prior to 2002 (represented by the horizontal dashed line), at which time the APA released its guidance for multicultural practice. Since that time, the greatest increases have been observed in reporting of race/ethnicity and SES and examining the psychometric properties of the study sample. We also saw improvements in avoiding lumping of minoritized racial groups into “other” categories.

## Discussion

The purpose of this study was to describe the extent to which EBPIs that have outcomes associated with child emotional/behavioral health and/or parenting include research methods, design, and interpretation strategies that are aligned with best practices for racial equity in research. Concerns associated with lack of relevance of EBPIs across populations, and particularly for minoritized and racialized populations, are well documented (Doyle et al., 2023; Weisenmuller & Hilton, 2021) and substantial focus has been placed on supporting adaptations of EBPIs for these populations. We posit that, in service to this noble goal, it is imperative for research and evaluation around these programs to be conducted in accordance with CRE best practices. We further believe that adhering to these best practices does not compromise, and may likely enhance, rigor. This study demonstrated that while there is some use of these approaches within the programs and services that meet evidentiary standards for FFPSA, there is substantial room for continued growth. We acknowledge that this study was conducted with a specific focus on programs with policy relevance in the United States. However, this general approach may be relevant to any global social policy that uses evidence to justify implementation at a population level. Research and evaluation activities necessarily happen within the specific context in which they are being conducted. Therefore, it is important to consider the implications for people who may not be represented in the research and identify strategies to enhance the external validity of studies.

### Utility of the Evaluation Framework in Research

The coding manual developed for the present study was an amalgamation of several different resources describing how to build racial equity into research, including best practices for conducting research and reporting findings. We hope that the coding manual (Supplemental Appendix I) will provide some guidance to researchers seeking to conduct culturally responsive and race equitable research through each stage, from design and execution through data analysis, interpretation, and dissemination. Considering strategies to address racial equity throughout the entirety of the research process can enhance the external validity of studies without compromising the internal validity of study designs.

Overall, we found the coding framework developed for this study to be feasible. The framework was used with a high degree of interrater reliability; discrepant codes were typically easily resolved through conversation. That said, our coding team was intentionally ‘generous’ in providing credit to studies for using racial equity domains and utilized a non-judgmental approach whereby we were simply exploring the degree to which various CRE practices were employed. Future studies could evaluate the extensiveness or completeness of studies in their incorporation of the various codes throughout the research process.

### Most Frequently Observed Racial Equity Domains

The most used racial equity-related approaches observed in the papers and programs reviewed for this study were related to analysis and reporting. This includes assessing the validation of measures for the study sample and reporting participant race/ethnicity and socioeconomic status. This is unsurprising as there has been clear guidance over the past twenty years indicating that this is the best practice for all social science research, not just CRE (Boyd et al., 2022; Cheng et al., 2015). Ensuring that study participants are representative of the intended service population was another technique that was relatively more common in our reviewed studies. Historically, there have been critiques of the evidence-based practice literature that studies consisted predominantly of middle-class white participants (Langer et al., 2021; Roberts et al., 2020). Our review of studies demonstrates that, at least for the studies of EBPIs designated as having evidentiary support by the Clearinghouse, study participants were frequently from diverse backgrounds and of varied socioeconomic statuses. This finding is aligned with a recent study of the Blueprints for Healthy Youth Development program registry as well, in which most studies reported race (Buckley et al., 2023). Across studies included in their review, 65% of the study sample consisted of participants identifying as non-White, though there were substantial inconsistencies in how race and ethnicity were reported (Buckley et al., 2023). Finally, it was relatively common for considerations related to diversity to be included in the conclusion sections of reviewed papers. Paper authors frequently reflected on the overall diversity of the study sample and how that generalizes (or does not) to other populations or concluded additional research was warranted to further understand the generalizability of findings.

### Most Infrequently Observed Racial Equity Domains

There were many best practices for racial equity that we attempted to find in the reviewed papers but were never (or only rarely) observed in our sample. Many of the codes that were less frequently observed were those that involved collaboration with community members. For example, use of CBPR/YBPR was nonexistent in our sample. This could also be a function of the type of research that is eligible for review by the Clearinghouse which limits eligibility to only randomized controlled trials (RCTs) and quasi-experimental designs (QEDs) (Wilson et al., 2019) where CBPR/YBPR approaches remain less common. However, conducting CBPR/YBPR does not preclude use of RCT or QED designs. For example, a recent paper describes the utility of CBPR RCTs in American Indian populations (Rink et al., 2020). Further, a recent systematic review indicates a substantial increase in the use of CBPR-based methods in recent clinical trials (McFarlane et al., 2022). This review of ten years of clinical trials revealed a four-fold increase in the number of CBPR-based studies, improvement in population representation, and in general positive treatment effects for around 85% of reviewed studies (McFarlane et al., 2022).

None of the studies indicated that they had trained evaluators on aspects of cultural competence or humility. The critical importance of ensuring that research staff, including evaluators, are trained and understand the unique cultural contexts of study participants has been extensively described (e.g., Skaff et al., 2002). It is possible that, for some of the studies reviewed, this was a component of research training that was not described in the manuscript. As it has been described as an ethical imperative for researchers to engage in culturally competent and responsive practice (Woodland et al., 2021), reporting of such training support can help the research field better understand strategies that improve research engagement and relevance for diverse populations.

None of the papers reviewed described using cognitive interviewing or pilot testing as a step to ensure participant understanding of measures. Study methods such as piloting test measures or identifying strategies to protect against differential attrition are CRE-informed strategies that enhance the internal validity of research studies. Nápoles-Springer et al. (2006) conducted a study that illustrates the value of cognitive interviewing with a racially and ethnically diverse sample. This study started with 159 potential survey items and closely looked at respondent behavior and conducted interviews. They found 20% of study items were very problematic, exceeding a 15% threshold for concern. A further 79% of items that were below that threshold still had some concerns across one or more problem areas; concerns differed by racial/ethnic group (Nápoles-Springer et al., 2006). In our own work, we have found that merely translating the language survey questionnaires may unintentionally introduce slight differences in interpretation (e.g., very much can be interpreted as too much) that impact responding.

We found only one paper and study that used specific retention efforts with the intention of reducing disproportionate attrition (Duggan et al., 2005). This paper described comprehensive efforts including supporting home visiting staff to gain trust, use of incentives, gathering information about friends or other supports who could help find the family within the research window if needed, and collaboration with the post office to notify of address changes. Of course, as aligned with best practices, many studies reported overall attrition, and compared attrition across conditions. However, taking the step to report if attrition varies by different participant characteristics is valuable. It is possible that overall differential attrition is minimal, but a closer look at within-condition attrition could reveal systematic differences. These differences could be related to intervention acceptability, research methods acceptability, or both. One study of depression treatment looked closely at this issue (Murphy et al., 2013). Study authors found engagement and treatment satisfaction were particularly important predictors of study retention for African Americans in the study sample.

Although still relatively infrequent across our paper sample, six papers representing three programs specifically included racial equity-related analyses as part of their primary research questions (Brief Strategic Family Therapy, Familias Unidas, and Family Check-Up), while two additional programs considered racial equity-related analyses post hoc (Multidimensional Family Therapy and Nurse Family Partnership). We were intentionally liberal in our application of this code, as our team wanted to capture any use of analyses that help differentiate what works for whom, including if programs work equally well across groups. All programs assessing racial equity as a primary research question and one that assessed racial equity post hoc met evidentiary requirements to achieve a “well-supported” designation in the Clearinghouse. Multidimensional Family Therapy, which explored such analyses post hoc, met criteria for “supported.” We did not further code the ways in which racial equity analyses were conducted, but this is an important area for future reviews. For example, to conduct racial equity analyses, care must be taken regarding how data are collected and how the population is understood within the context of the research (Edmonds et al., 2021). Given we also infrequently observed use of strategies such as having participants self-describe their race/ethnicity and gender and rarely encountered consideration of place within analysis, it is not surprising that relatively few of the studies reviewed were positioned to be able to conduct racial equity analyses using best practices.

Only one study (of the Video Interaction Project) looked at intersectionality of race with other dimensions of identity. Intersectionality recognizes the ways that systemic inequalities related to different social identities often intersect to compound an individual’s experience of these inequalities (Crenshaw, 1991). This overall lack of consideration of intersectionality in evidence-based programming is of concern given that the dynamic intersection of determinants with a participant’s identity is touted as the main causes leading to health disparities. Previously mentioned factors such as poverty/lack of resources, limited access to high-quality health care, adverse life experiences, systems of discrimination, and oppression often interact with gender, race, LGBTQI + status, and ability status placing certain groups at higher risk of negative outcomes (Biglan et al., 2023; Hawn et al., 2022). The lack of consideration of intersectionality can play out in a variety of ways for evidence-based programs including (1) excluding certain groups from intervention based on disability status, (2) underestimating the logistical challenges in accessing the intervention, (3) dismissing or ignoring major environmental constraints such as toxic physical environments; harmful media and marketing exposure and diminished neighborhood and school quality, and (4) minimizing the impact of trauma experienced by marginalized groups (women, people of color, LGBTQI +) as a result of system of discrimination (Biglan et al., 2023).

To help ameliorate the issues associated with the impact of intersectionality on outcomes in evidence-based practice, Biglan et al. (2023) highlight that not only do interventions need to be designed to be more comprehensive but public policy also needs to change. Currently, US-based policies tend to focus prevention efforts more on risk factors and evaluation of single programmatic interventions and not as much on health disparity and equity. Biglan et al. (2023) propose shifting research focus to experimental evaluation of multiple interventions and the evaluation of comprehensive community interventions that simultaneously address risk factors and determinants of health disparities. With this approach, the detrimental impact of intersectionality on outcomes can be potentially reduced across domains.

Finally, we note that although many papers included considerations of diversity in the conclusions section, we did not observe any papers that specifically noted inclusion of community groups or program participants in the crafting of the conclusions and implications. Including community members at this stage is a best practice within CBPR and the CRE model, though it is one of the least utilized approaches (Cashman et al., 2008). Involving community members in the dissemination of research findings is similarly important. McDavitt and colleagues describe several core strategies that can be used to ensure a transparent and supportive process, including having a flexible dissemination plan, tailoring presentations to the different audiences, having a community liaison, and following up after presentations. They further highlight the importance of intentionally building rapport and trust within communities toward implementing these strategies (McDavitt et al., 2016).

### Studies Conducted Outside of the United States

Several of our coded studies were conducted outside of the United States, including Ecuador and Iceland. Our team debated retaining these studies or not, given the unique sociopolitical climates and demographic characteristics of these countries. We worried inclusion could result in conflating macro-level differences with our study aims. However, upon reflection, we decided to retain the studies because the research supporting the programs was relevant to US-based policy decisions; indeed, the Clearinghouse did not exclude them. These studies still had opportunities to incorporate racial equity-related principles in their designs. This does raise the question of whether our coding scheme is robust in its consideration of the cultural and racial diversity in international samples, when the macro-level contexts are different. This is an area we will explore more fully in future studies.

### Changes Over Time

Some modest increases in use of racial equity-informed research approaches were observed for several coding domains, and it appears that many of these shifts happened after the specific guidance from the APA in 2002. We note, however, that the largest changes are in areas specific to reporting, and less so in areas that represent deeper incorporation of CRE such as involving community members, conducting racial equity-related analyses, and examining constructs associated with place and racism and oppression. We hope that with the recent presidential executive order (Exec. Order No. 13985, 2021), there will be an even greater emphasis on incorporating racial equity within research, and this will be encouraged by Federal funding agencies. Our findings reveal that there is a promising foundation but still a lot of work to be done in terms of consistently approaching research with a strong equity lens.

### Limitations

There are several limitations to our current study that are important to acknowledge. First, this was the first time that this article coding system was used. As such, we used a more developmental process to confirm codes and adjusted some of the language based on coder feedback. It is likely further refinement would be valuable to support future efforts using this coding strategy. The inclusionary criteria used for the present study limited the scope of articles reviewed. The criteria were chosen due to the focus of the special issue and the Clearinghouse was used to identify papers because of the policy relevance of programs included on the Clearinghouse registry. As such, the findings should be considered applicable only to this very narrow set of programs and services, and the studies associated with their evidence base as defined by the Clearinghouse. Further studies are needed to increase the generalizability of findings. Finally, while it is possible that programs have additional studies that have been completed after the review by the Clearinghouse, resource limitations precluded searching for additional studies.

### Practical Implications of the Findings

In this section, Dr. Chaundrissa Oyeshiku Smith, one of Dr. Prinz’s doctoral students, who is currently a child/adolescent clinical psychologist and program director in a large Health Maintenance Organization (HMO) in the Southeast United States, reflects on the practical implications of this study.

In a clinical decision-maker role, it is critical that there is evidence that programs and services adequately address the needs of children and families. An important component of this is the extent to which these programs and services are relevant to a diverse patient population. Knowing that studies incorporate values aligned with racial equity helps increase confidence in the overall evidence base—it’s not enough to simply know that programs ‘work.’ We must understand what works for whom and under what circumstances. One way to help disentangle this is to embed racial equity practices within research. We cannot take for granted that because a body of evidence exists around a program’s effectiveness that it is necessarily applicable or translatable to another service setting, community, or population. As someone who supports practitioners to make everyday decisions about what services are best for particular clients, helping support their use of research knowledge and increasing their confidence in the validity and relevance of the studies is important. This study shows that there have been some modest improvements in the incorporation of racial equity approaches in research. If this trend continues, it will greatly help build transparency and confidence in the research literature.

### Call to Action

This review highlights ways in which rigorous research can incorporate racial equity into the planning, design, execution, and interpretation and dissemination of the programs of study. Doing so improves the external validity of studies while maintaining high-quality research that can contribute to an evidence base. Our call to action is for intervention researchers and evaluators to use frameworks that prioritize race equity and culturally responsiveness, such as CRE, when conducting studies. The evidence-based practice field has come far in the past thirty years, in gratitude to researchers such as Drs. Prinz and Ollendick. As we more comprehensively consider how to meet population needs, attending to racial equity in studies is essential. Although not specific to parenting interventions, we highlight a recent article by Burlew et al. (2021) that outlines many practical steps to meaningfully incorporate racial/ethnic equity within research and the Multicultural Orientation Framework (Raque et al., 2021) as two additional resources in addition to the CRE framework (among many recently emerging approaches) that outline a path forward.

### Supplementary Information

Below is the link to the electronic supplementary material.

Supplementary file1 (DOCX 42 KB)
