# Supplementary material for: An Equity-Focused Assessment of Evidence-Based Parenting Intervention Research
Source: Clin Child Fam Psychol Rev. 2024 May 16;27(2):279–99. doi: 10.1007/s10567-024-00479-2 (PMC11222220; doi:10.1007/s10567-024-00479-2)
Supplement: Supplementary file 1 — Supplementary file1 (DOCX 42 KB) [file 10567_2024_479_MOESM1_ESM.docx]

**Parenting Programs and Racial Equity Coding Manual**

**Version 3 (8/25/2023)**

**Eligibility:**

- Programs must be:
  - In-Home Parent Skill-Based Program on the Prevention Services Clearinghouse
  - Well-Supported, Supported, or Promising rating
  - Each study that is included must have an outcome associated with Child well-being: behavioral and emotional functioning AND Adult well-being: Positive parenting practices
  - Rated as of July 11, 2023
- Studies must:
  - Have a rating as high or moderate (therefore included in the rating). Cannot have a rating of low or not eligible for review)

**Background assumptions and definitions:**

- All elements are coded 0 or 1, with 0=no and 1=yes. For those that are unable to be determined,
  - If you do not observe a domain, code as 0
  - If you are unsure, code as 0, but indicate ‘difficult to code’ in notes section
  - Use comment boxes in the survey for items that may require further discussion during inter-rater reliability
- If necessary, err on the side of inclusion (if there is any evidence that domains were considered).
- Each study will be assigned a number (consistent with study number in the PSC). Subsequent papers associated with each study get a paper number. For example, if the study is number 14246, the first paper is 14246.1, the second paper is 14246.2 and so on.
- Code all domains, even if race/ethnicity isn’t included and thus many other codes would be ‘no’, proceed with coding all domains.

**___________________________________________________________________**

**CODING**

**___________________________________________________________________**

**Background**

**Program/Service demographic information: (Consult the Clearinghouse)**

- Primary target age range (include all that apply) (choices 0-18; emerging adulthood)
- Prevention or treatment program
  - Prevention: The primary purpose of the intervention is to either prevent adverse outcomes, or address risk and strengthen protective factors before a disorder or problem emerges.
  - Treatment: The primary purpose of the intervention is to address concerns that have already emerged (e.g., behavior problems, emotional disorders)
- Target population:
  - Target need areas (open ended): List the areas that the intervention is designed to address (e.g., disorders, problem areas, risk factors)
  - Parent target population features (open ended): List all areas that the intervention specifically focuses on. This must be listed in the target population tab on the Clearinghouse website or be described in the general program description. For consistency, please write in the following order to describe the population (for any categories that apply):
    - Race/ethnicity
    - Marital Status (e.g., single mothers)
    - Parent/caregiver status (e.g., mothers, fathers, grandparents, kinship, foster, other; first time mothers)
    - Living situation (e.g., multigenerational)
    - Geographic focus (e.g., rural, Tribal Nation)
    - Socioeconomic status (e.g., all Medicaid; free and reduced lunch eligible)
  - Child/youth population features (open ended), may be the same as above
- Program’s available languages (check all that apply)
  - English
  - Spanish
  - Other: List
- PSC Rating (check one)
  - Well-supported
  - Supported
  - Promising
- Total number of studies constituting the evidence rating. Count the number of studies receiving a moderate or high rating. (on Clearinghouse page for the program, go to “Extent of Evidence” and add up the numbers in “Rated High” and “Rated Moderate” rows)
- Total number of studies reviewed by the Clearinghouse. Count all studies reviewed, regardless of rating, and including does not meet.
- Number of studies exceeded 10 (yes/no). The reason for this rating is that if a program or service reaches a well-supported rating within 10 studies, other studies are only reviewed for risk of harm, so they wouldn’t be included and could impact the breadth of studies that are included in our review. Look in Clearinghouse under the program “Extent of Evidence” tab at the number of studies that are eligible for review. If more than 10, select yes.

**Paper Demographic information**

- - Type of document (select one)
    - Peer-reviewed manuscript. The paper is in a peer-reviewed journal.
    - U.S.-based Governmental report (including grant reports). Paper is a grant report or other source that was commissioned by a U.S.-based governmental body
    - Non-U.S.-based Governmental report (including grant reports). Paper is a grant report or other source that was commissioned by a governmental body outside of the United States
    - Commissioned report. The paper was published by a non-Governmental, non-profit, for-profit, or other entity. E.g., Annie E. Casey Foundation
    - Other (e.g., non-commissioned report) (Specify)
  - Type of study (select one)
    - RCT. Study design randomizes people to conditions
    - QED. Study design uses quasi-experimental methods (e.g., propensity score matching)
  - Outcome domains studied. Go to INDIVIDUAL STUDY FINDINGS tab on the Clearinghouse website and select all that apply
    - Child Safety
    - Child Permanency
    - Child well-being: Behavioral and Emotional Functioning
    - Child well-being: Social functioning
    - Child well-being: Cognitive Functions and Abilities
    - Child well-being: Educational Achievement and Attainment
    - Child well-being: Physical Development and Health
    - Child well-being: Substance use or misuse
    - Child well-being: Delinquent Behaviors
    - Adult well-being: Parenting Practices
    - Adult well-being: Parent/Caregiver Mental or Emotional Health
    - Adult well-being: Parent/Caregiver Substance Use or Misuse
    - Adult well-being: Parent/Caregiver Criminal Behavior
    - Adult well-being: Family Functioning
    - Adult well-being: Physical Health
    - Adult well-being: Economic and Housing Stability
  - Who were the study respondents? Review the methods section and indicate yes for any measures that were administered to the following (select all that apply):
    - Child report
    - Parent/Caregiver report
    - Teacher report
    - Biological or physiological measurement
    - Administrative data
    - Observation data (e.g., parent-child interactions)
    - Other (specify)

**_____________________________________________________________________________________**

**RACIAL and ETHNIC EQUITY DOMAINS**

The following domains are coded as Yes or No for whether there is evidence that any of these factors are in the papers reviewed. If it is unclear, do your best to rate the items but note any challenges in the open-ended response options at the end.

As you are reviewing each paper, highlight where in the paper you find the evidence for each code. This will help if we need to reconcile differences between reviewers and enable quick confirmation of codes.

**Research Planning/Study Development**

**CBPR/YBPR or other community engagement techniques.** Does the paper describe any use Community-Based Participatory methods or other strategies to engage community members in any aspect of the paper? This could include design, administration, data analysis, and/or data interpretation. Use the notes field to briefly describe. Using community-based therapists or simply giving gift cards or other renumeration does not count for this code.

**Research question/s directly relate to racial equity.** Do any of the research questions (primary or secondary, or post-hoc) relate to racial or ethnic equity broadly defined? Examples could include looking at disproportionality, differences between groups to ensure the intervention has equal effectiveness, differential dropout, family engagement/intervention acceptability, etc.

**Evaluator training.** Study authors described training of evaluators in the best practices and nuances in working with the population of focus for the study (Select N/A for purely administrative data studies). Note if there is information about evaluator status (e.g., research assistant, grad student, post-doc, permanent employee)

**Race/ethnicity in the study.** In the introduction, methods or discussion section, the study authors described how they define race and why/how it was used in the study.
(Select 'N/A' for those studies that did not report on race)

**Methods and Analysis Planning**

**Mixed methods or stories.** Quantitative data were supplemented with qualitative data to contextualize quantitative data. This could be a formal mixed methods approach, or it could be in the form of specific quotes or stories that were included.

**Racial Equity Analyses.** Note if any RE-specific analyses were conducted (even if not part of the primary or secondar research questions). The authors must state that the purpose of the analysis was to explore, understand, or otherwise describe aspects relevant to racial and/or ethnic equity.

**Participant identification.** The paper explicitly states, or there is clear evidence, that participants were recruited in ways that were likely to include representative members of society (e.g., purposive sampling techniques to ensure proportional racial inclusion are described) or sampling of specific groups for which the intervention is designed (e.g., program is for AIAN populations, so there is a match with recruiting to ensure the population of interest is included). Code yes if any such methods were used, regardless of whether the authors directly linked the strategy to racial equity goals.

**Participant retention.** There is evidence that the paper authors engaged in retention efforts with the intention of reducing disproportionate attrition. If yes, describe the participant retention efforts.

**Used cognitive interviewing or pilot testing on measures.** Cognitive testing helps researchers understand how different people interpret questions. Indicate ‘yes’ if there was a step to ensure that the research measures were applicable to the individuals responding to the measure.

**Validation or reliability metrics.** Indicate yes if authors reported on psychometric properties *at all* for any of the measures in the paper. If yes…

**Metrics of the population in the paper.** Indicate yes if authors reported *paper specific* psychometric properties for measures, showing that they are valid and/or reliable for the specific population under study. Select no if they relied on reporting for prior validation/reliability studies or they did not report psychometrics at all.

**Oversampling.** Authors described efforts to purposefully oversample populations that have relatively smaller representation so to avoid making conclusions based on small sample sizes.

**Correlated place to outcomes.** The paper included features of participant place (e.g., census block, zip code, neighborhood) in examining outcomes. This may be described as a strategy that enables looking at the role of historical policies in interpreting findings.

**Social Determinants.** The paper measures explicitly included perceptions of discrimination or oppression.

**Data Reporting**

**Race/ethnicity.** Indicate yes if the paper included any information about participant racial or ethnic demographic characteristics. Select no if the paper did not provide any information about race/ethnicity of participants (if no here, then skip the next two)

**Self-describe race/ethnicity.** Participants were able to self-describe their race, and/or ethnicity. This means that there were no pre-defined options; participants could identify however they wanted. If primary participants were young children (under 12), their parents were able to describe their race/ethnicity. Choose N/A if the authors do not report race/ethnicity at all.

If no, was there an option for participants (child or parent) to write in their race if a drop-down menu was provided?

**Self-describe gender.** Participants were able to self-describe their gender. This means that there were no pre-defined options; participants could identify however they wanted. If primary participants were young children, their parents were able to describe their gender. Choose N/A if the authors do not report gender at all.

If no, was there an option for participants (child or parent) to their gender if a drop-down menu was provided?

**Use of “other”.** In the analysis plan or results, racial/ethnic groups with small n’s were lumped together and characterized as “other.” Choose N/A if they do not report race/ethnicity at all.

**CONSORT included race/ethnicity.** The paper clearly described how participants moved through the study and where there was attrition AND race/ethnicity was included. OR the paper clearly described (but didn’t have a CONSORT diagram) where participants were lost to the analysis and included race/ethnicity information. Choose N/A if they do not report race/ethnicity at all. If they did give a description of race/ethnicity but did not report it in the results (CONSORT or description) then “no”.

**Racial disaggregation.** Paper authors provided results separately by race or ethnicity. Choose N/A if they do not report race/ethnicity at all.

**Differential attrition.** The paper examined attrition and if differential attrition occurred by race/ethnicity. Choose N/A if they do not report race/ethnicity at all.

**Sub-group analysis.** The paper included subgroup analyses by race/ethnicity and/or other areas of intersectionality to describe what works for whom. Choose N/A if they do not report race/ethnicity at all.

**SES data.** Data are presented on the Socioeconomic status of program participants (either primary data collection or imputed)

**Program acceptability.** The paper reported on the acceptability of the intervention to participants, disaggregated by race/ethnicity. Choose N/A if they do not report race/ethnicity at all.

**Intersectionality of race with other dimensions of identity.** Analysis plan included examining the intersectionality of race with other dimensions of identity on study outcomes or research questions. Examples of areas of intersectionality include LGBTQIA+, disabilities, immigrants and people with limited English, unstably housed, history of incarceration, substance use disorders, survivors, mental health disorders (Bledsoe & Londhe, 2022). Choose N/A if they do not report race/ethnicity at all.

**Racial equity analyses prominence.** Racial equity-related analyses were conducted as either

1. Analyses were part of primary research questions
2. Analyses related to race or racial equity were conducted as post-hoc analyses
3. Not part of analyses
4. Other: describe

**Paper conclusions and interpretations**

**Included community groups for support for data interpretation.** The paper specifically indicated that results were shared with community groups (or others outside of the study group who represent those who participated in the study) to get support for data interpretation.

**Indicated limitations associated with racial equity.** The paper had at least one limitation listed that mentioned race/ethnicity. This could be a limitation describing what was not able to be assessed given the data limitations or related to interpretation of findings.

**Acknowledged structural bias.** The paper authors noted that structural racism or other harms could impact the communities that are part of the data and/or potentially be an explanatory factor.

**Conclusions.** At least one of the conclusions drawn by the paper focused on a feature of diversity, inclusion, or equity.

**Other**

**Difficult to code**

Please note here if there were any domains that were particularly difficult to code.

**Racial-equity or related framework (open ended)**

Note if authors specified a RE or related framework; describe.

**Notes**

In this section, please note any other observations you had about the paper as it relates to racial equity.
